# Impact of preoperative anemia and perioperative transfusion on short‐term outcomes in colorectal cancer surgery: The role of iron supplementation

**DOI:** 10.1002/ags3.12867

**Published:** 2024-10-04

**Authors:** Junpei Takashima, Hirotoshi Kobayashi, Ayaka Koizumi, Fumi Shigehara, Kenji Yamazaki, Daisuke Fujimoto, Fumihiko Miura

**Affiliations:** ^1^ Department of Surgery Teikyo University School of Medicine, Mizonokuchi Hospital Kawasaki Kanagawa Japan

**Keywords:** colorectal cancer, ferrous citrate, hemoglobin, iron‐deficiency anemia, oral iron supplementation

## Abstract

**Background and Aim:**

Colorectal cancer is a common malignancy, and many patients with colorectal cancer experience preoperative anemia. Anemia and transfusions negatively impact short‐term surgical outcomes. Management of anemia, including iron supplementation, has not been extensively studied in Japanese patients. Thus, the impact of anemia and blood transfusions on short‐term surgical outcomes in colorectal cancer patients and the effectiveness of oral iron supplementation with ferrous citrate were investigated.

**Methods:**

A retrospective study of patients with colorectal cancer (≥18 y) who underwent elective surgery from April 2015 to March 2023 was conducted. Patients with benign tumors, malignant lymphoma, emergency surgeries, or nonresectable lesions were excluded from the study. Hemoglobin levels were assessed at consultation, admission, the day after surgery, and discharge. Patients were categorized by anemia severity and divided into iron supplementation and no supplementation groups. Outcomes, including transfusions and postoperative complications, were compared with univariate and multivariate analyses.

**Results:**

The prevalence of postoperative anemia in the 545 enrolled patients increased significantly from 52.8% at admission to 78.7% the day after surgery (*p* < 0.001). Severe anemia immediately before surgery was an independent risk factor for postoperative complications (odds ratio [OR] = 9.24, *p* < 0.001). Iron supplementation significantly improved hemoglobin levels and reduced transfusions and complications. The median duration of iron supplementation was 30 d, suggesting a positive influence on outcomes.

**Conclusion:**

Severe anemia immediately before surgery is an independent risk factor for postoperative complications. Iron supplementation with ferrous citrate improves short‐term outcomes.

## 1 INTRODUCTION

Colorectal cancer is among the most prevalent malignancies globally. In 2020, colorectal cancer was the third most common cancer in men and the second most common cancer in women, affecting ~1.9 million people.^1^ In Japan, around 160 000 people were diagnosed with colorectal cancer in 2019, making it the second most common cancer among both men and women.^2^ Many patients with colorectal cancer suffer from anemia before surgery. The prevalence of anemia in patients with colorectal cancer is ~48%, and 25% of the cases are moderate to severe anemia.^3^, ^4^ Preoperative anemia adversely impacts short‐term outcomes such as postoperative complications and perioperative mortality. Thus, preoperative anemia is an independent prognostic factor for poor outcomes.^5^ Even mild anemia is associated with prolonged hospital stays and increased complications.^6^ The long‐term implications of anemia include decreased disease‐free survival, higher overall mortality, and increased cancer‐related deaths and surgical site infections, although these associations remain controversial.^7^, ^8^

The treatment for moderate to severe anemia typically involves red blood cell transfusion. However, transfusions may be an independent prognostic factor for adverse outcomes.^9^ The mechanisms underlying the association between transfusions and adverse outcomes are unclear, but may involve immunosuppression associated with increased postoperative infections.^10^ Consequently, the World Health Organization (WHO) advocates for patient blood management and supports a restrictive transfusion approach in preoperative surgical management.^11^

Iron supplementation is a viable alternative to transfusion. Iron‐deficiency is the primary cause of anemia in patients with colorectal cancer, affecting up to 88% of cases.^12^ Oral iron supplementation is the standard treatment for iron‐deficiency anemia and is an effective treatment before colorectal cancer surgery.^13^ Furthermore, iron‐deficiency may be a prognostic factor for poor outcomes in colorectal cancer,^14^ underscoring the appropriateness of preoperative iron supplementation. Intravenous iron administration has also been reported to be effective and offers benefits over oral iron supplementation, such as fewer gastrointestinal side effects and faster iron absorption.^15^, ^16^ However, most of these studies were conducted outside Japan using ferrous sulfate. In Japan, ferrous citrate, which is absorbed better than ferrous sulfate, is commonly used. Only a few studies focused on the impact of anemia and blood transfusions on colorectal cancer treatment in Japanese patients. The lack of relevant studies highlights the need for research on the effectiveness of iron supplementation therapy with ferrous citrate. We routinely administer oral ferrous citrate to patients with iron‐deficiency anemia at our institution and have observed the effectiveness of this therapy. This study aimed to explore the effectiveness of preoperative ferrous citrate in improving short‐term surgical outcomes in patients with colorectal cancer.

## 2 MATERIALS AND METHODS

### 2.1 Study design and participants

This was a retrospective study of consecutive colorectal cancer cases diagnosed and operated on at our institution from April 2015 to March 2023. Patients ≥18 y old with confirmed or suspected diagnoses of colorectal cancer who underwent elective surgery were included in the study. Patients diagnosed with benign tumors or malignant lymphoma postoperatively, patients requiring emergency surgery, and patients with nonresectable primary lesions were excluded from the study.

### 2.2 Data collection and evaluation

We assessed demographic information and short‐term surgical outcomes, including procedure, operative time, blood loss, postoperative complications (Clavien–Dindo grade II or higher), and length of postoperative hospital stay. Hemoglobin levels were evaluated at consultation (Pe1), at admission (Pe2), the day after surgery (Pe3), and at discharge (Pe4). Anemia was defined according to the WHO standards as follows: Mild: Hb = 11–11.9 g/dL in females and 12–12.9 g/dL in males; Moderate: Hb = 8.0–10.9 g/dL in females and 9.0–11.9 g/dL in males; or Severe: Hb ≤7.9 g/dL in females and ≤8.9 g/dL in males.

### 2.3 Risk factors for postoperative complications

Patients were divided into complication and no complication groups based on the presence or absence of postoperative complications, respectively. Patient backgrounds and perioperative factors were retrospectively analyzed to identify risk factors for postoperative complications.

### 2.4 Effectiveness of iron supplementation

Patients were divided into groups with and without iron supplementation. Patient backgrounds, changes in hemoglobin levels, the presence of preoperative, intraoperative, and postoperative transfusions, and the presence of complications were compared between the two groups. Predictors of increased hemoglobin were investigated in the iron supplementation group. The decision to administer iron supplementation at our institution was left to the discretion of the attending physician, with no specific criteria.

### 2.5 Statistical analysis

Statistical analyses were performed using EZR.^17^ Normal distribution was evaluated using the Shapiro–Wilk test. Nonnormally distributed numerical variables were analyzed using the Mann–Whitney *U* test. Categorical variables were analyzed using the chi‐square or Fisher's exact tests, as necessary. Multivariate logistic regression analyses were conducted to assess the association between anemia or transfusion and the incidence of postoperative complications and the association between iron supplementation and changes in hemoglobin levels. Variables for inclusion were selected based on the results of univariate analyses and their clinical significance. A *p*‐value <0.05 was considered statistically significant. Numerical data are presented as medians and ranges (minimum‐maximum).

### 2.6 Ethical considerations

This study was approved by the Research Ethics Committee of Teikyo University (approval no. 20–049) and was conducted as a retrospective analysis of existing medical records in accordance with the guidelines for the protection of personal information.

## 3 RESULTS

The study included 545 patients, consisting of 321 males and 224 females. The median age was 73 y. Detailed information about lesion locations and surgical methods is shown in Table 1. Right‐sided lesions accounted for 42.2% of cases. Open surgery was performed in only 9.2% of cases, with a conversion rate to open surgery of 2% for laparoscopic and robotic surgeries. The incidence of postoperative complications of Clavien–Dindo grade 2 or higher was 12.8%, and the mortality rate within 30 d postsurgery was 0.6% (three cases).

**TABLE 1:** Patient backgrounds and perioperative results.

Variables	All patients
	*n* = 545
Sex^b^	
Male	321 (58.9)
Female	224 (41.1)
Age (y)^a^	73 (30–101)
Body mass index (kg/m^2^)^a^	22.1 (14.1–41.2)
ASA‐PS Class 1/2/3/4	35/422/87/1
Serum albumin level (g/dL)^a^	3.8 (1.4–5.4)
Prognostic nutritional index^a^	44.7 (31.2–67.8)
Comorbidity^b^	
Total	447 (82.0)
Diabetes	118 (21.7)
Hypertension	240 (44.0)
Heart disease	117 (21.5)
Respiratory disease	77 (14.1)
Cerebrovascular disease	58 (10.6)
CEA (ng/mL)^a^	4.2 (0.5–105)
CA19‐9 (U/mL)^a^	4.9 (2.0–708)
Preoperative anemia^b^	288 (52.8)
Iron supplementation^b^	91 (16.7)
Lesion location^b^	
Cecum	75 (13.8)
Ascending colon	110 (20.1)
Transverse colon	45 (8.3)
Descending colon	31 (5.7)
Sigmoid colon	111 (20.4)
Rectosigmoid	62 (11.4)
Upper rectum	62 (11.4)
Lower rectum	49 (8.9)
Surgical approach^b^	
Laparotomy	50 (9.2)
Laparoscopic surgery	411 (75.4)
Robotic surgery	84 (15.4)
Conversion to laparotomy^b^	10 (2.0)
Surgical procedures^b^	
Ileocecal resection	108 (19.8)
Right hemicolectomy	102 (18.7)
Left hemicolectomy	20 (3.7)
Partial colectomy	132 (24.2)
High anterior resection	53 (9.7)
Low anterior resection	78 (14.3)
Hartmann	28 (5.1)
Abdominoperineal resection of the rectum	21 (3.9)
Total pelvic exenteration	3 (0.6)
Dissection D1/D2/D3	12/84/449
Surgical curability R0/R1/R2	483/8/54
Operation time (min)^a^	241 (66–1183)
Blood loss (mL)^a^	25 (0–2750)
Blood transfusion^b^	
Preoperative	34 (6.2)
Intraoperative and postoperative	70 (12.8)
Postoperative hospital stay (d)^a^	9 (5–85)
Complication^b^	70 (12.8)
Pneumonia	12 (2.2)
Anastomotic leakage	9 (1.7)
Intestinal obstruction	9 (1.7)
Ileus	9 (1.7)
Urinary tract infection	3 (0.6)
Surgical site infection	3 (0.6)
T factor 1/2/3/4	85/63/262/135
Stage I/II/III/IV	121/196/149/79
Mortality cases within 30 d postsurgery^b^	3 (0.6)

Abbreviations: ASA‐PS, American Society of Anesthesiologists Physical Status Classification; CA19‐9, carbohydrate antigen 19–9; CEA, carcinoembryonic antigen.

^a^ Median (range).

^b^ *n* (%).

### 3.1 Hemoglobin values and anemia status

**FIGURE 1:**
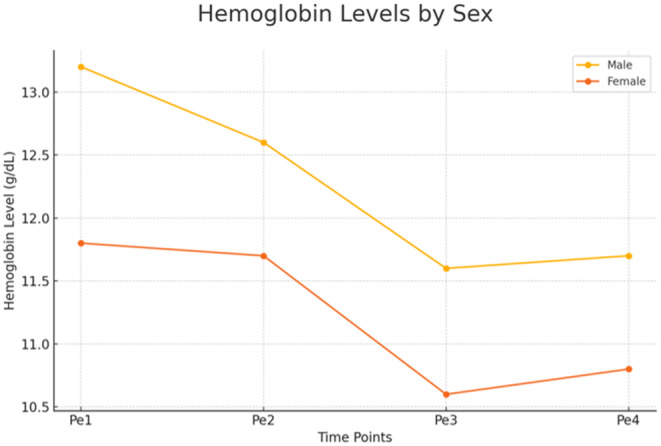
Hemoglobin levels by sex across different timepoints.

**FIGURE 2:**
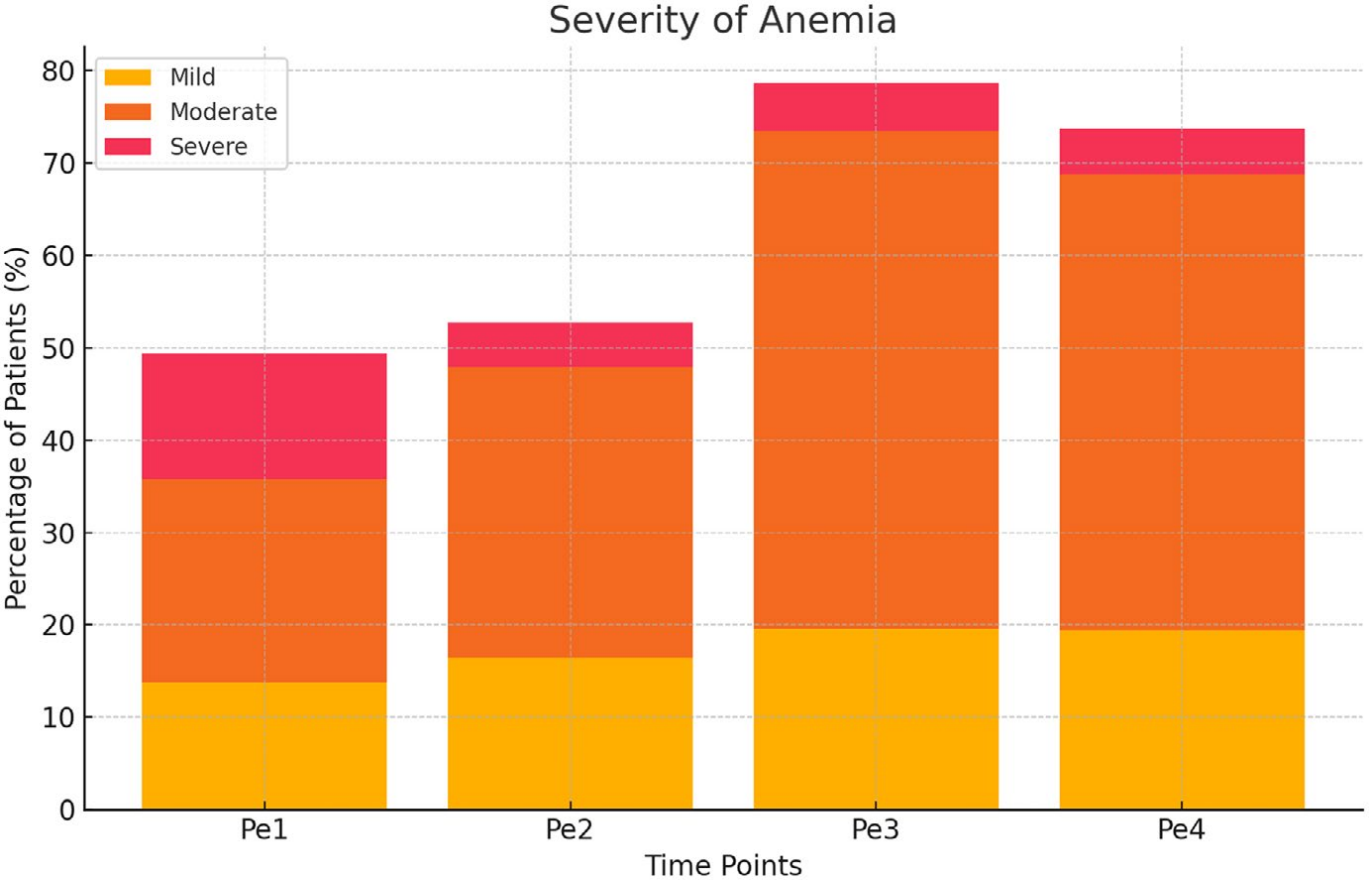
Severity of anemia at different timepoints.

The perioperative hemoglobin values are shown in Figure 1, and the severity of anemia is shown in Figure 2. The median time from initial consultation to surgery was 26 d (range: 5–246 d). The prevalence of total anemia was 49.4% at consultation (Pe1) and 52.8% at admission (Pe2) (*p* = 0.364) and did not change significantly over time. However, the prevalence of severe anemia significantly decreased from 13.6% at consultation (Pe1) to 4.9% at admission (Pe2) (*p* < 0.001). The prevalence of total anemia increased significantly postoperatively to 78.7% on the day after surgery (Pe3) and 73.8% at discharge (Pe4) (*p* < 0.001 for both comparisons to preoperative values).

### 3.2 Anemia‐related background factors

Univariate analyses comparing background factors according to anemia severity at consultation (Pe1) are shown in Table 2. The prevalence of anemia was 46.4% (149/321) in males and 53.6% (120/224) in females. Patients in the anemia groups exhibited significantly lower serum iron and ferritin levels, serum albumin values, and prognostic nutritional index (PNI) and were older. The incidence of circumferential lesions was significantly higher across all severities of anemia. T factors and stage were more advanced in the anemia group, particularly in cases of moderate and severe anemia. Anemia was more prevalent in patients with right‐sided colorectal cancer and increased with anemia severity. Preoperative transfusions were not performed in cases of mild anemia and were only performed in 2.5% of moderate anemia cases. However, preoperative transfusions were performed in 41.9% of severe anemia cases. In contrast, iron supplementation was more common in mild cases.

**TABLE 2:** Analysis of risk factors contributing to anemia at consultation (Pe1).

Variables	No anemia	Total anemia	*p*‐value	Mild anemia	Moderate anemia	Severe anemia
	*n* = 276	*n* = 269		*n* = 75	*n* = 120	*n* = 74
Sex^b^			0.12			
Male	172 (62.3)	149 (55.4)		44 (58.7)	65 (45.2)	40 (54.1)
Female	104 (37.7)	120 (44.6)		31 (41.3)	55 (45.8)	34 (45.9)
Age (y)^a^	69 (31–93)	78 (30–101)	<0.01	72 (34–98)	79 (42–96)	79 (30–101)
Serum albumin level (g/dL)^a^	4.0 (2.1–5.4)	3.4 (1.4–4.7)	<0.01	3.6 (1.4–4.7)	3.4 (1.6–4.7)	3.2 (1.6–4.4)
Prognostic nutritional index^a^	48.6 (32.9–67.8)	41.3 (31.2–57.8)	<0.01	44.4 (34.5–59)	40.2 (33.1–55.3)	39.3 (31.2–52.5)
ASA‐PS Class 1/2/3/4	27/213/36/0	8/209/51/1	<0.01	3/62/10/0	3/88/29/0	2/59/12/1
Hemoglobin level^a^						
Male	14.5 (13–17.4)	10.8 (4.3–12.9)	<0.01	12.5 (12–12.9)	10.6 (9–11.9)	7.6 (4.3–8.9)
Female	13.4 (12–15.6)	9.5 (4.5–11.9)	<0.01	11.5 (11–11.9)	9.6 (8–10.9)	7.1 (4.5–7.9)
Serum iron^a^	71 (18–246)	20.5 (6–162)	<0.01	45 (17–136)	28 (7–162)	13 (6–127)
Serum ferritin^a^	93.9 (5.7–515.9)	16.0 (4.9–606.2)	<0.01	55.6 (7.5–362.2)	16.6 (4.9–606.2)	10.5 (4.9–168.6)
Comorbidity^b^	218 (79)	229 (85.1)	0.07	65 (86.7)	104 (86.7)	60 (81.1)
Iron supplementation^b^	1 (0.4)	90 (33.5)	<0.01	3 (4.0)	44 (36.7)	43 (58.1)
Circumferential lesion^b^	63 (22.9)	113 (42.3)	<0.01	25 (33.8)	46 (38.7)	42 (56.8)
Colorectal stent^b^	26 (9.4)	25 (9.3)	0.97	11 (14.7)	6 (5)	8 (10.8)
Preoperative blood transfusion^b^	0	34 (12.6)	<0.01	0	3 (2.5)	31 (41.9)
Lesion location: right sidedness	93 (33.7)	130 (48.3)	<0.01	29 (38.7)	59 (49.2)	42 (56.8)
T factor 1/2/3/4	58/38/124/56	27/25/138/79	<0.01	9/10/43/13	16/10/58/36	2/5/37/30
Stage I/II/III/IV	80/89/75/32	41/107/74/47	<0.01	15/32/19/9	22/47/31/20	4/28/24/18

Abbreviation: ASA‐PS, American Society of Anesthesiologists Physical Status Classification.

^a^ Median (range).

^b^ *n* (%).

Anemia at Pe1 was significantly associated with factors such as advanced age, lower PNI, and more advanced tumor stage, indicating its role as a marker of poor preoperative health and nutritional status.

### 3.3 Risk factors for postoperative complications

Univariate analyses between the complication and no complication groups are shown in Table 3. No significant differences in preoperative comorbidities were detected between the two groups. Complications were more frequent in patients with lesions deeper than T3, but stages were not different between the groups. Significant differences were detected in age, serum albumin levels, PNI, circumferential lesions, open surgery, creation of stoma, operation time, blood loss, postoperative hospital stay, and preoperative, intraoperative, and postoperative transfusions. Significant differences between the complication and no complication groups at Pe2 were detected in patients with moderate and severe anemia, and differences between the complication and no complication groups at Pe1, Pe3, and Pe4 were detected in patients with severe anemia. Stepwise multivariate analysis identified severe anemia at Pe2 (odds ratio [OR] = 9.24, *p* < 0.01), preoperative transfusion (OR = 4.05, *p* < 0.01), intraoperative/postoperative transfusion (OR = 12.8, *p* < 0.01), and the creation of stoma (OR = 2.93, *p* = 0.04) as independent predictors of postoperative complications. Postoperative hospital stay was not included in the multivariate analysis because it was considered an outcome variable rather than a risk factor.

**TABLE 3:** Analysis of risk factors for postoperative complications.

Variables	Complication group	No complication group	*p*‐value	OR	95% Cl	*p*‐value
	*n* = 70	*n* = 475				
Sex male/female	42/28	279/196	0.9			
Age (y)^a^	78 (42–101)	72 (30–98)	<0.01	1.02	0.98–1.06	0.35
Body mass index (kg/m^2^)^a^	21.9 (14.1–33.2)	22.1 (15.1–41.2)	0.14			
Serum albumin level (g/dL)^a^	3.4 (1.6–4.8)	3.8 (1.4–5.4)	<0.01	1.19	0.21–6.76	0.84
Prognostic nutritional index^a^	39.5 (31.2–67.8)	45.4 (32.8–65.8)	<0.01	1.03	0.91–1.17	0.61
ASA‐PS Class 1/2/3/4	3/58/8/1	32/364/79/0	0.3			
Comorbidity^b^	63 (90)	384 (80.8)	0.09			
At presentation (Pe1) Hb^b^						
Total anemia^b^	49 (70)	218 (45.9)	<0.01	0.78	0.35–1.72	0.53
Mild anemia^b^	6 (8.6)	66 (13.9)	0.81			
Moderate anemia^b^	14 (20)	106 (22.3)	0.18			
Severe anemia^b^	29 (41.4)	45 (9.7)	<0.01	1.41	0.43–4.61	0.57
Preoperative (Pe2) Hb^b^						
Total anemia^b^	51 (72.9)	237 (49.9)	<0.01	1.09	0.5–2.35	0.828
Mild anemia^b^	7 (10)	83 (17.5)	0.99			
Moderate anemia^b^	24 (34.3)	147 (30.9)	0.03			
Severe anemia^b^	20 (28.6)	7 (1.5)	<0.01	9.24	2.59–33	<0.01
On postoperative day 1 (Pe3) Hb^b^						
Total anemia^b^	57 (81.4)	372 (78.3)	0.64			
Mild anemia^b^	8 (11.4)	99 (20.8)	0.37			
Moderate anemia^b^	38 (54.3)	256 (53.9)	0.74			
Severe anemia^b^	11 (15.7)	17 (3.6)	<0.01	1.01	0.18–5.55	0.99
At discharge (Pe4) Hb^b^						
Total anemia^b^	59 (84.3)	343 (72.2)	0.04	0.68	0.29–1.6	0.37
Mild anemia^b^	11 (15.7)	95 (20)	0.5			
Moderate anemia^b^	39 (55.7)	230 (48.4)	0.06			
Severe anemia^b^	9 (12.9)	18 (3.8)	<0.01	1.33	0.18–10.1	0.78
Iron supplementation^b^	13 (18.6)	78 (16.4)	0.61			
Circumferential lesion^b^	17 (21.5)	42 (51.2)	<0.01	1.66	0.53–5.21	0.38
Colorectal stent^b^	9 (12.9)	42 (8.8)	0.27			
Surgical procedures: laparotomy^b^	16 (22.9)	34 (7.2)	<0.01	0.32	0.04–2.7	0.29
Conversion to laparotomy^b^	2 (2.8)	6 (1.7)	0.09			
Dissection D1/D2/D3	3/13/54	9/71/395	0.22			
Stoma	23 (32.9)	48 (10.1)	<0.01	2.93	1.01–8.53	0.04
Operation time (min)^a^	291.5 (86–1008)	247 (66–1183)	<0.01	1.03	0.95–1.12	0.4
Blood loss (mL)^a^	139 (0–2750)	20 (0–1430)	<0.01	1.01	0.91–1.11	0.87
Blood transfusion^b^						
Preoperative	17 (24.3)	17 (3.6)	<0.01	4.05	2.1–7.8	<0.01
Intraoperative and postoperative	39 (55.7)	31 (6.5)	<0.01	12.8	8.5–19.3	<0.01
T factor T3 or T4	56 (80)	341 (71.8)	0.08			
Stage I/II/III/IV	5/26/22/17	116/170/127/62	0.1			
Postoperative hospital stay (day)^a^	25 (7–61)	9 (5–85)	<0.01			

Abbreviations: ASA‐PS, American Society of Anesthesiologists Physical Status Classification; Hb, hemoglobin.

^a^ Median (range).

^b^ *n* (%).

### 3.4 Effectiveness of iron supplementation

The anemia groups were subdivided into iron supplementation and no iron supplementation groups, and univariate analyses of changes in hemoglobin values were performed in the subgroups (Table 4). Hemoglobin levels at Pe2, Pe3, and Pe4 increased significantly compared with hemoglobin levels at Pe1 in the iron supplementation group. In the severe anemia group, preoperative and intraoperative/postoperative transfusions and complications rates were significantly lower and postoperative hospital stays were significantly shortened in the iron supplementation group compared with the no iron supplementation group.

**TABLE 4:** Effects of iron supplementation.

Variables	Total anemia		*p*‐value	Mild anemia		*p*‐value	Moderate anemia		*p*‐value	Severe anemia		p‐value
	Iron supplementation	No iron supplementation		Iron supplementation	No iron supplementation		Iron supplementation	No iron supplementation		Iron supplementation	No iron supplementation	
	*n* = 90	*n* = 179		*n* = 3	*n* = 72		*n* = 44	*n* = 76		*n* = 43	*n* = 31	
Sex male/female	38/52	111/68	<0.01	0/3	44/28	0.07	17/27	48/28	0.01	21/22	19/12	0.35
Age (y)^a^	80 (30–101)	77 (34–98)	<0.01	84 (83–96)	72 (34–98)	<0.01	79 (42–96)	79 (42–96)	<0.01	78 (30–101)	80 (47–89)	0.93
Hemoglobin level^a^												
Male	8.6 (5.1–11.5)	11.3 (4.3–12.9)	<0.01	–	12.5 (12–12.9)	–	9.8 (9.2–11.5)	11 (9–11.9)	<0.01	7.5 (5.1–8.9)	7.9 (4.3–8.9)	0.30
Female	8.2 (4.5–11.4)	10.7 (4.6–11.9)	<0.01	11.2 (11.2–11.4)	11.5 (11–11.9)	0.23	8.9 (8–10.9)	10.3 (8.6–10.9)	<0.01	7.2 (4.5–7.9)	6.7 (4.6–7.9)	0.53
Changes in hemoglobin levels												
From Pe1 to Pe2	+1.8 (−0.9 ± 6.4)	0 (−0.2.5 ± 6.2)	<0.01	0 (0 ± 0.6)	−0.2 (−2.5 ± 1.0)	0.09	+1.35 (−0.9 ± 3.7)	0 (−2.3 ± 2.8)	<0.01	+3.0 (+0.1 ± 6.4)	+1.8 (−1.3 ± 6.2)	<0.01
From Pe1 to Pe3	+1.3 (−3.8 ± 6.8)	−1.0 (−6.6 ± 6.3)	<0.01	−0.9 (−0.9 to −0.3)	−1.5 (−6.6 ± 0.3)	0.09	+0.45 (−3.8 ± 2.9)	−0.9 (−2.6 ± 4.5)	<0.01	+2.7 (−0.6 ± 6.8)	+2.5 (−2.1 ± 6.3)	0.89
From Pe1 to Pe4	+1.9 (−1.2 ± 7.4)	−0.8 (−6.6 ± 6.8)	<0.01	−1.0 (−1.2 to −0.9)	−1.4 (−6.6 ± 0.7)	0.47	+0.75 (−1.1 ± 3.0)	−0.8 (−3.2 ± 4.2)	<0.01	+3.1 (+0.2 ± 7.4)	+2.4 (−1.2 ± 6.8)	0.14
Serum iron^a^	16 (7–60)	29 (6–136)	<0.01	22 (22–40)	47 (17–136)	0.09	24 (7–56)	30 (14–97)	0.09	13 (7–60)	16 (6–127)	0.11
Serum ferritin^a^	13.6 (4.9–98.1)	24.8 (4.9–606.2)	<0.01	55.6 (8–10)	42.2 (7.5–362.2)	0.87	15.7 (4.9–89.5)	23.7 (5–606.2)	0.05	10.1 (4.9–98.1)	12.8 (4.9–76.9)	0.38
Blood transfusion^b^												
Preoperative	13 (14.4)	21 (11.7)	0.56	0	0	1	0	3 (3.9)	0.3	13 (30.2)	18 (58.1)	0.02
Intraoperative and postoperative	20 (22.2)	39 (21.8)	0.97	1 (33.3)	5 (6.9)	0.22	8 (18.2)	14 (18.4)	0.97	11 (25.6)	20 (64.5)	<0.01
Postoperative hospital stay (day)^a^	9 (6–101)	10 (6–85)	0.07	10 (8–10)	10 (6–54)	0.67	9 (6–52)	11 (6–85)	0.04	10 (7–36)	21 (7–61)	<0.01
Complication^b^	13 (14.4)	36 (20.1)	0.32	1 (33.3)	5 (6.9)	0.22	4 (9.1)	10 (13.2)	0.57	8 (18.6)	21 (67.7)	<0.01

Abbreviations: ASA‐PS, American Society of Anesthesiologists Physical Status Classification; Pe1, at consultation; Pe2, at admission; Pe3, the day after surgery; Pe4, at discharge.

^a^ Median (range).

^b^ *n* (%).

Iron supplementation effectively improved hemoglobin levels and reduced the need for blood transfusions, especially in patients with severe anemia, suggesting that it improves short‐term outcomes.

### 3.5 Predictors of increased hemoglobin

**FIGURE 3:**
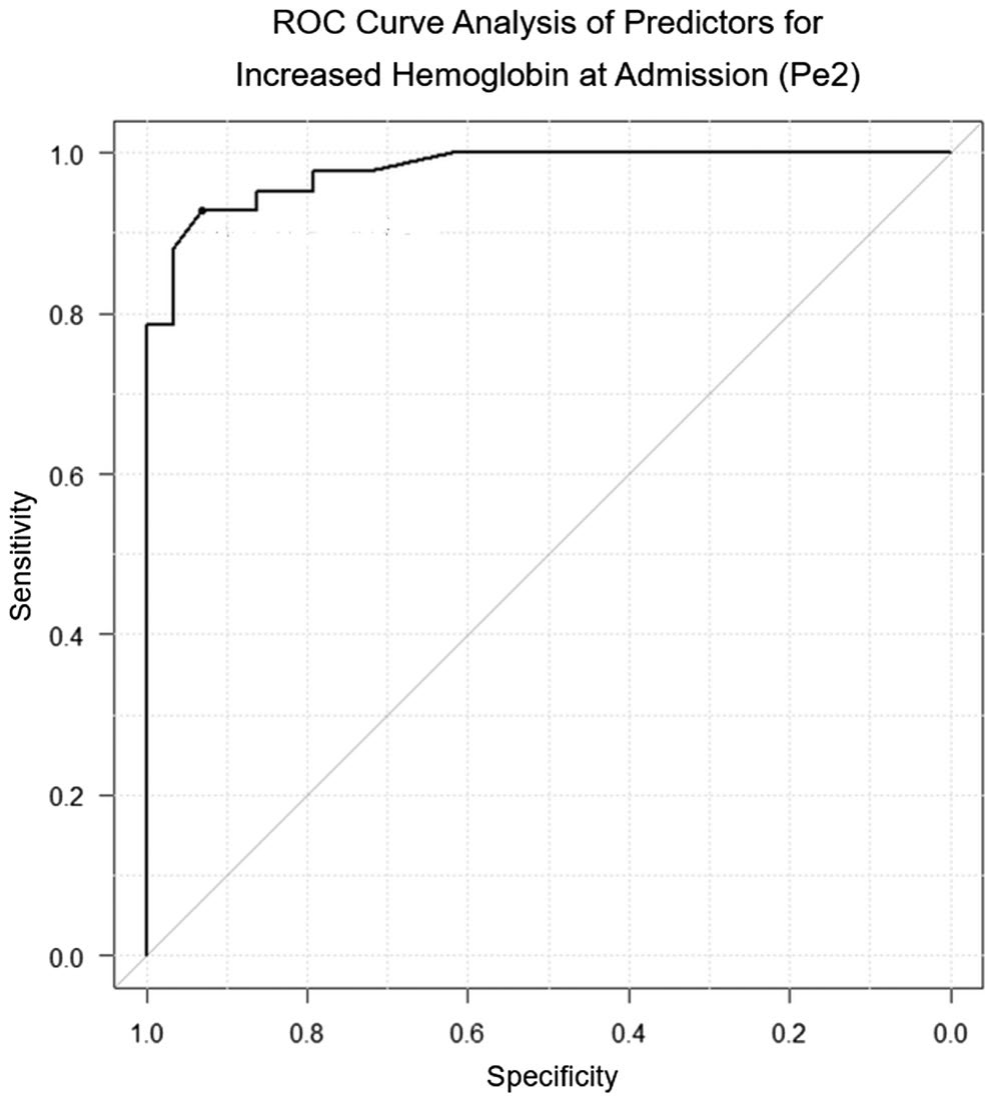
ROC curve analysis of predictors for increased hemoglobin at admission (Pe2).

Patients in the iron supplementation group (78 of 91 patients) who did not receive preoperative transfusions were analyzed to identify predictors of increased hemoglobin. An Hb increase of 1.5 g/dL was adopted as the criterion for treatment efficacy in iron‐deficiency anemia. Patients were divided into hemoglobin increase and no increase groups measured from Pe1 to Pe2 based on this criterion. Serum iron levels, serum ferritin levels, the duration of iron supplementation, and the degree of hemoglobin increase after 2 weeks of iron supplementation were significant predictors of increased hemoglobin levels in univariate analyses (Table 5). Stepwise multivariate analysis revealed that the degree of hemoglobin increase after 2 weeks of iron supplementation (OR = 235, *p* = 0.001) was the only independent predictor of increased hemoglobin. A receiver operating characteristic (ROC) curve analysis based on this value indicated a cutoff of +0.8 g/dL (AUC = 0.978, sensitivity 0.931, and specificity 0.929), demonstrating a very high predictive accuracy (Figure 3). Although the duration of iron supplementation was not an independent risk factor, ROC curve analysis revealed a cutoff value of 30 d (AUC, 0.726, sensitivity, 0.676, and specificity, 0.750).

**TABLE 5:** Predictors of increased hemoglobin at admission (Pe2).

Variables	Univariate analysis			Multivariate analysis		
	Hb improved group	Hb unchanged group	*p*‐value	OR	95% Cl	*p*‐value
	*n* = 44	*n* = 34				
Serum iron^a^	14 (7–50)	37 (7–60)	<0.01	0.95	0.85–1.05	0.32
Serum ferritin^a^	9.8 (4.9–98.1)	18.7 (5.7–89.5)	<0.01	0.97	0.90–1.13	0.85
Duration of iron supplementation^a^	42.5 (8–108)	20 (4–208)	<0.01	1.06	0.98–1.15	0.12
Hb change after 2w of iron supplementation^a^	+1.6 (+0.2 ± 3.3)	+0.1 (−0.7 ± 1.2)	<0.01	235	9.1–6080	<0.01

Abbreviations: 2w, 2 weeks; Hb, hemoglobin.

^a^ Median (range).

The degree of hemoglobin increase after 2 weeks of iron supplementation was identified as the only significant predictor of treatment response, underscoring the importance of early hemoglobin response monitoring in the management of preoperative anemia.

## 4 DISCUSSION

Unlike previous studies, this study focused on changes in hemoglobin levels from the time of consultation to just before surgery, the day after surgery, and discharge. Preoperative anemia was identified as a negative prognostic factor for outcomes in colorectal cancer surgery.^5^, ^6^, ^7^, ^8^ However, by evaluating hemoglobin levels separately at the time of consultation and just before surgery, our study demonstrated that severe anemia immediately before surgery is an independent risk factor for postoperative complications. This finding underscores the importance of managing anemia from the time of initial consultation. Additionally, our results confirm that transfusions at any point, including the preoperative, intraoperative, or postoperative periods, were associated with an increase in complications. This result supports the WHO's recommendations for patient blood management, highlighting the need for conservative transfusion practices and the consideration of alternative treatments.

Our results demonstrate that iron supplementation is highly effective in correcting iron‐deficiency anemia. Furthermore, iron supplementation significantly reduced postoperative complications in patients with severe anemia and improved hemoglobin levels in patients with moderate to severe anemia. This analysis included patients who underwent transfusions. Thus, the influence of transfusions on hemoglobin levels was considered. The numbers of preoperative, intraoperative, and postoperative transfusions were comparable between the iron supplementation and no supplementation groups in patients with moderate anemia. However, in the severe anemia group, patients in the iron supplementation group underwent fewer transfusions compared with patients in the no supplementation group. Nevertheless, the hemoglobin levels were elevated, indicating that iron supplementation effectively increased hemoglobin levels. The reduced number of transfusions in the severe anemia group suggests that iron supplementation is an effective treatment option to avoid transfusions.

The outcomes of patients who received iron supplementation in this study are promising compared to other studies, suggesting that the method of oral iron supplementation practiced at our institution favorably impacts short‐term outcomes. One reason for this favorable impact may be the prolonged duration of iron supplementation. The average duration of iron supplementation in other studies was 14 d^13^ or at least 14 d,^18^ whereas the median duration was 30 d (range: 4–208 d) in our study. ROC curve analysis for the duration of iron supplementation revealed a cutoff value of 30 d (AUC = 0.726), suggesting that this duration is statistically sufficient and the longer supplementation period may have contributed to favorable outcomes.

Additionally, the use of ferrous citrate as an iron supplement is noteworthy. Recent studies have examined whether oral or intravenous administration of iron is more effective. From the perspective of adverse events, intravenous iron supplementation was superior.^15^ A meta‐analysis also demonstrated that oral iron supplementation is associated with gastrointestinal symptoms, including constipation and diarrhea.^16^ However, at our hospital no adverse event was reported and no patient discontinued the oral medication. This may be due to the fact that ferrous sulfate was used as the iron supplement and that the meta‐analysis predominantly involved noncancer patients (including many patients with inflammatory bowel disease), with only 2 out of the 23 studies focusing on patients with cancer.^19^, ^20^ The adverse events associated with oral iron supplementation are considered dose‐dependent.^16^ While ferrous sulfate is typically administered at 325 mg twice or three times a day, ferrous citrate is administered at 200 mg/day. From a dosage perspective, ferrous citrate may be superior in terms of fewer adverse events. We considered that the absence of adverse events and the ability to continue treatment with ferrous citrate may have contributed to the favorable outcomes.

Based on the results of this study, we also discuss the route of administration. It is said that oral iron administration requires more time for absorption. In patients scheduled for colorectal surgery, the absorption rate from oral administration may be insufficient to quickly restore hemoglobin levels and reposition iron deposits.^15^ To address this issue, we ensured a longer administration period than previously reported, resulting in higher hemoglobin levels, similar to the levels reported with intravenous administration.^15^, ^16^ Even with oral administration, long‐term use of ferrous citrate, as practiced at our hospital, may provide results comparable to intravenous administration. The appropriate method of iron administration should consider cost, the risk of side effects, and the urgency of treatment, but oral administration using ferrous citrate may also be a viable option.

A protocol for iron supplementation has not been established at our hospital. Based on this study, we propose rapidly introducing oral ferrous citrate at 200 mg/day for patients with iron‐deficiency anemia diagnosed at the initial consultation and evaluating the change in hemoglobin levels after 2 weeks. If hemoglobin levels do not increase by 0.8 g/dL or more, transitioning to intravenous iron supplementation should be considered. Transfusions should be limited to cases of severe, symptomatic anemia.

The generalizability of the results of this study may be limited because it was a retrospective study conducted at a single institution. To further verify the efficacy and safety of the proposed iron supplementation protocol, multi‐institutional prospective studies are needed. Future research should compare ferrous citrate with ferrous sulfate and oral versus intravenous administration.

## 5 CONCLUSION

Severe anemia immediately before surgery is an independent risk factor for postoperative complications. Preoperative transfusions were detrimental, but the use of iron supplements for anemia treatment may be beneficial. Our findings indicate that oral iron supplementation using ferrous citrate is promising, but further studies are needed to establish best practices, including the potential use of intravenous iron administration.

## AUTHOR CONTRIBUTIONS

**Junpei Takashima:** Conceptualization; data curation; formal analysis; investigation; methodology; project administration; writing – original draft; writing – review and editing. **Fumihiko Miura:** Conceptualization; formal analysis; writing – review and editing. **Daisuke Fujimoto:** Conceptualization; formal analysis; writing – review and editing. **Ayaka Koizumi:** Data curation; investigation; visualization. **Kenji Yamazaki:** Data curation; investigation; project administration; visualization. **Fumi Shigehara:** Data curation; investigation; project administration.

## FUNDING INFORMATION

No funding was received.

## CONFLICT OF INTEREST STATEMENT

The authors declare no conflict of interest for this article.

## ETHICS STATEMENT

This study was approved by the Teikyo University Medical Research Ethics Committee (approval no. 20–049) and performed according to the Declaration of Helsinki (as revised in Fortaleza, Brazil, October 2013). Written informed consent was obtained from all individual participants included in the study.

## Data Availability

All data generated or analyzed during this study are included in this published article.
